# Reliability of the Test of Visual Perceptual Skills-4th Edition for people with schizophrenia

**DOI:** 10.1371/journal.pone.0318148

**Published:** 2025-03-04

**Authors:** Shu-Chun Lee, En-Chi Chiu

**Affiliations:** 1 Department of Occupational Therapy, Taipei City Psychiatric Center, Taipei City Hospital, Taipei, Taiwan; 2 Department of Recreation and Sports Management, University of Taipei, Taipei, Taiwan; 3 Department of Long-Term Care, National Taipei University of Nursing and Health Sciences, Taipei, Taiwan; King Faisal University, SAUDI ARABIA

## Abstract

**Background:**

The Test of Visual Perceptual Skills-4th Edition (TVPS-4) is widely used for repeated measures of visual perception. This study aimed to examine the test-retest reliability, internal consistency, and practice effect of the TVPS-4 in people with schizophrenia receiving care in community psychiatric rehabilitation facilities.

**Methods:**

A repeated assessment design was employed, involving 80 participants. Test-retest reliability was evaluated using the intraclass correlation coefficient (ICC) and Pearson’s *r*. Internal consistency was evaluated using Cronbach’s alpha (α). Minimal detectable change (MDC) values were calculated at 95%, 90%, and 80% confidence levels. The MDC% was determined based on the MDC with 95% certainty. Cohen’s *d* effect sizes and paired *t*-tests were utilized to assess the practice effect.

**Results:**

The ICC and Pearson’s *r* of the overall scale were 0.93 and 0.95, respectively. The ICC and Pearson’s *r* of the seven subscales were 0.59–0.84 and 0.61–0.84, respectively. The α was 0.66–0.90 and 0.73–0.89 in the first and second assessment, respectively. The MDC_95_ (MDC%), MDC_90_, and MDC_80_ ranged from 4.7–16.3 (13.2–34.8%), 3.9–13.6, and 3.1–10.6, respectively. Cohen’s *d*s were 0.06–0.26 and paired *t*-test showed significant differences in scores of the overall scale and the four subscales (*p* <  0.05).

**Conclusion:**

The TVPS-4 has acceptable test-retest reliability, satisfactory internal consistency, and trivial to small practice effect. The MDCs at different confidence levels can be used to interpret the score changes at a particular level of certainty for individuals with schizophrenia.

## Introduction

Visual perception, also referred to as visual information processing, encompasses the cognitive processes involved in recognizing and interpreting visual stimuli in the environment, integrating visual information with other sensory systems, and generating appropriate responses [1, 2]. More than 60% of people with schizophrenia have visual perceptual deficits [3]. A previous study using functional magnetic resonance imaging showed impaired functioning of the visual pathway in people with schizophrenia [4]. The clinical manifestations of visual perceptual deficits in those with schizophrenia include difficulties in object discrimination, impaired perception of the entirety of objects, and challenges in distinguishing foreground from background elements [5–7]. Visual perceptual deficits affect their performance of functional activities [8]. Therefore, clinicians and researchers should use a visual perceptual measure to comprehend visual perception conditions, formulate related treatment plans, and track treatment progress effectively for people with schizophrenia.

The Test of Visual Perceptual Skills-4th Edition (TVPS-4) is a motor-free visual perceptual measure [9] and an updated version of the widely used TVPS-3, which assesses visual perception in adults [10, 11]. The TVPS-4 contains seven subtests: visual discrimination, visual memory, spatial relationships, form constancy, sequential memory, visual figure-ground, and visual closure. The TVPS-4 retains the items of the TVPS-3 and adds two items with lower item difficulty in each subtest, which is suitable for people with severe visual perception deficits. The scoring method for the TVPS-4 has been modified to discontinue a subtest when examinees obtain five out of seven items incorrectly [2]. The TVPS-4 has three following features. First, assessing visual perception requires perception, not the ability to reproduce perception through the motor process. Second, the easy-to-read design with only pictures raises the usefulness of this measure for people with cognitive impairments. Third, it has multiple subtests to assess diverse domains of visual perception, which is profitable for defining the strengths and weaknesses of visual perception comprehensively. Thus, the TVPS-4 is an adequate measure for assessing visual perception in people with schizophrenia in both clinical and research settings.

Temporal stability and internal consistency are essential psychometric properties of scores obtained from visual perceptual measures. Test-retest reliability estimates whether scores obtained from a measure yields consistent results over repeated assessments [12]. Internal consistency reflects how reliably items within a measure produce consistent results [13]. Random measurement error, an unpredictable factor affecting test results, should be taken into consideration when interpreting score changes. Minimal detectable change (MDC) refers to the minimum threshold to verify whether a score change of a person between repeated assessments is far from the random measurement error at a particular confidence level (e.g., 95%, 90%, and 80%) [14]. A score change below the MDC threshold indicates that it may be attributed to random measurement error. The practice effect is described as improvements in test results over repeated assessments owing to previous experiences in administration of the same measure [15]. To ensure meaningful interpretation of test results in both clinical and research settings, it is essential to examine test-retest reliability, internal consistency, and practice effect.

The TVPS-3 presented acceptable test-retest reliability and good internal consistency in people with stroke [16]. However, the test-retest reliability, internal consistency, and practice effect have not been investigated in previous editions nor the TVPS-4 for people with schizophrenia. Therefore, this study aimed to examine the test-retest reliability, internal consistency, and practice effect of the TVPS-4 in people with schizophrenia.

## Methods

### Participants

A total of eighty people with chronic schizophrenia were recruited from one psychiatric center in northern Taiwan between January 4th, 2021 and June 22nd, 2022. The inclusion criteria were as follows: (1) diagnosis of schizophrenia based on the Diagnostic and Statistical Manual of Mental Disorders, fifth edition; (2) age over 20 years; (3) stable drug compliance and adherence; and (4) willingness to participate in this study. The exclusion criteria were (1) diagnosis of brain injury and intellectual developmental disorders; (2) visual impairment; and (3) unstable psychiatric symptoms (with inconsistent scores on the Clinical Global Impressions-Severity scale [CGI-S] in two assessments). This study was approved by the research ethics committee of Taipei City Hospital (TCHIRB-10905018). Written informed consent was obtained from all participants before administering the assessments.

The eighty participants completed the two assessments and 42.5% were men, with an average age of 43.8 years. The average age of onset age for schizophrenia was 21.2 years. The details of the participants are presented in Table 1.

**Table 1:** Demographics of participants (N = 80).

Characteristic	N (%), mean (SD)
Gender	
Male	34 (42.5)
Female	46 (57.5)
Age, year	43.8 (11.3)
Onset age, year	21.2 (6.8)
Education	
Elementary and below	1 (1.3)
Junior high school	6 (7.5)
Senior high school	33 (41.3)
College and above	40 (50.0)
Marital status	
Single	71 (88.8)
Married	5 (6.3)
Other	4 (5.0)
CGI-S	2.9 (0.6)

SD: standard deviation; CGI-S: Clinical Global Impressions Scale-Severity subscale.

### Procedures

One examiner administered the TVPS-4 twice with a two-week interval for each individual participant who met the recruitment criteria. The inclusion and exclusion criteria were assessed through a thorough evaluation of each participant’s medical history and current condition by the examiner. The examiner received at least 3 hours of training to familiarize herself with the measures (the test instructions, items, and scoring) and practiced administering the measures on two people with schizophrenia. The first author observed and conducted training for the examiner to ensure consistent and reliable assessments. According to the design of the TVPS-4, the subscales were conducted in the order of visual discrimination, visual memory, spatial relationships, form constancy, sequential memory, visual figure-ground, and visual closure. All participants were assessed in a quiet environment to avoid disturbance. On average, 3 to 5 participants were examined per day. The demographic data of the participants were gathered from medical charts.

### Measures

The TVPS-4 assesses visual perception using seven subscales. In the visual discrimination subscale, participants are required to point one picture out of five that matches the target. In the visual memory subscale, the participants first memorize the target and then point it out on the next page. In the spatial relationships subscale, the participants point out a picture that is different from the others. In the form constancy subscale, the participants point out a picture that is similar to the target, regardless of any changes in size or position. In the sequential memory subscale, the participants memorize targets and their sequences and then point them out on the next page. In the visual figure-ground subscale, the participants point out the target that appears in different backgrounds. In the visual closure subscale, the participants point out a partial and incomplete picture of a complete target. Each subscale includes two practice items and 18 test items. Each item is rated as 0 (incorrect) or 1 (correct). The sum score (range 0-18) of the test items in each subtest represents the specific subscale function. The total score of the seven subtests (range 0-126) demonstrates general visual perception. A higher score indicates better visual perceptual ability [2].

The CGI-S is a clinician-rated scale that assesses the severity of psychiatric illness on a 7-point scale (1, no illness; 2, borderline illness; 3, mild illness; 4, moderate illness; 5, markedly ill; 6, severe illness; and 7, extremely severe illness). In this study, the CGI-S score was rated based on the examiner’s observation of the participants’ behaviors and self-reported symptoms. The score reflects the average severity level within 1 week [17, 18]. Sufficient convergent validity has been shown for the CGI-S in people with schizophrenia [19].

### Data analysis

For examining test-retest reliability, intraclass correlation coefficient (ICC) and Pearson’s *r* were calculated. The criteria for ICC and Pearson’s *r* were as follows: ≤  0.39, poor reliability; 0.40–0.59, moderate reliability; 0.60–0.79, good reliability; and ≥  0.80, excellent reliability [20, 21]. For examining internal consistency, Cronbach’s alpha (α) was utilized. The standard of coefficient α was >  0.70 [13]. The MDC was estimated by multiplying the standard error of the measurement (SEM) by the z score and $\sqrt{\text{2}}$ . The z score for the 95%, 90%, and 80% confidence interval were 1.96, 1.645, and 1.282, respectively. The SEM was computed by multiplying the standard deviation of the first assessment by $\sqrt{\text{1-ICC}}$ [14]. The MDC% was calculated by dividing the MDC_95_ by the maximum score and multiplied by 100. An MDC% <  30% represented an acceptable level of random measurement error [22].

We used effect size (Cohen’s *d*) to compute the extent of the practice effect due to repeated assessments. The Cohen’s *d* criteria were as follows: <  0.20, trivial effect size; 0.20–0.49, small effect size; 0.50–0.79, moderate effect size; and ≥  0.80, large effect size [23]. A paired *t*-*t*est (two-tailed, α=0.05) was applied to evaluate whether there was a significant difference between the two assessments.

## Results

Table 2 displays the results of test-retest reliability and internal consistency for the TVPS-4. The ICC and Pearson’s *r* were 0.93 and 0.95 for the overall scale of the TVPS-4, respectively. The ICC and Pearson’s *r* values of the subscales were 0.59–0.84 and 0.61–0.84, respectively. The Cronbach’s α of the seven subscales in the first and second assessment were 0.66–0.90 and 0.73–0.89, respectively. The MDC_95_ (MDC%) for the overall scale was 16.3 (13.2%) (Table 3). The MDC_90_ and MDC_80_ were 13.6 and 10.6, respectively. The MDC_95_, MDC_90_, and MDC_80_ of the seven subscales were 4.7–6.3, 3.9–5.3, and 3.1–4.1, respectively. With the exception of two subscales (visual discrimination and form constancy), the MDC% of the other subscales were <  30%.

**Table 2:** Test-retest reliability and internal consistency of the TVPS-4 (n = 80).

Scale and subscales	Mean_1_ (SD_1_)	Mean_2_ (SD_2_)	ICC (95% CI)	Pearson’s *r*	Cronbach’s α^a^
Overall scale	85.1 (21.8)	89.4 (22.3)	0.93 (0.83, 0.96)	0.95	–
Visual discrimination	11.3 (3.6)	12.1 (3.7)	0.63 (0.48, 0.75)	0.65	0.81/0.84
Visual memory	13.2 (2.8)	13.9 (2.8)	0.59 (0.42, 0.72)	0.61	0.66/0.73
Spatial relationships	14.4 (4.2)	14.8 (3.8)	0.83 (0.74, 0.89)	0.83	0.90/0.89
Form constancy	10.5 (4.5)	11.3 (4.4)	0.75 (0.63, 0.83)	0.76	0.88/0.88
Sequential memory	13.6 (3.1)	13.8 (3.1)	0.70 (0.56, 0.79)	0.69	0.77/0.78
Visual figure-ground	10.1 (4.3)	11.2 (4.4)	0.80 (0.67, 0.87)	0.82	0.87/0.88
Visual closure	12.1 (4.3)	12.4 (4.1)	0.84 (0.77, 0.90)	0.84	0.87/0.86

SD: standard deviation; ICC: intraclass correlation efficient.

^a^First assessment/second assessment.

**Table 3:** Minimal detectable change and practice effect of the TVPS-4 (n = 80).

Scale and subscales	SEM	MDC_95_ (MDC%)	MDC_90_	MDC_80_	Cohen’s *d*	Paired *t*-test (*p* value)
Overall scale	5.9	16.3 (13.2%)	13.6	10.6	0.19	−5.21 (<0.001)*
Visual discrimination	2.2	6.0 (33.6%)	5.1	4.0	0.22	−2.32 (0.023)*
Visual memory	1.8	4.9 (27.1%)	4.1	3.2	0.26	−2.66 (0.009)*
Spatial relationships	1.7	4.8 (26.7%)	4.0	3.1	0.10	−1.54 (0.128)
Form constancy	2.3	6.3 (34.8%)	5.3	4.1	0.19	−2.42 (0.018)*
Sequential memory	1.7	4.7 (26.3%)	4.0	3.1	0.06	−0.70 (0.489)
Visual figure-ground	1.9	5.3 (29.7%)	4.5	3.5	0.24	−3.48 (0.001)*
Visual closure	1.7	4.7 (26.1%)	3.9	3.1	0.06	−1.05 (0.299)

SEM: standard error of measurement; MDC: minimal detectable change.

*Significant at *p* <  0.05.

The Cohen’s *d* for the overall scale of the TVPS-4 was 0.19 (Table 2). The *d* values of the three subscales (visual discrimination, visual memory, and visual figure-ground) were >  0.20 and the *d* values of the other four subscales were 0.06–0.19. The paired *t*-*t*est showed statistically significant differences between repeated assessments for the overall scale and four subscales (visual discrimination, visual memory, form constancy, and visual figure-ground) (*p* <  0.05). No significant differences were found (*p* = 0.128–0.489) in the remaining three subscales.

## Discussion

To the best of our knowledge, this is the first study to examine the test-retest reliability, internal consistency, and practice effect of the TVPS-4 in people with schizophrenia. Our findings demonstrated excellent test-retest reliability and trivial practice effect for the overall scale. The TVPS-4 subscales revealed moderate to excellent test-retest reliability, sufficient internal consistency, and trivial to small practice effects. These results provide a solid foundation for the use of the TVPS-4 as a reliable assessment tool for visual perceptual skills in people with schizophrenia.

Four subscales of the TVPS-4 (form constancy, sequential memory, visual figure-ground, and visual closure) showed much more reliable ICC values (ICC = 0.70-0.84) in people with schizophrenia compared to those of the TVPS-3 (ICC = 0.55–0.77) in people with stroke [16]. These findings indicate that these four subscales demonstrated better test-retest reliability in assessing specific visual perceptual abilities for people with schizophrenia compared to the TVPS-3 in people with stroke, supporting its applicability in the schizophrenia population. The visual memory subscale showed relatively lower coefficient values of test-retest reliability and internal consistency, which were similar to reliability results of the TVPS-3 in people with stroke [16]. A possible reason for the lower reliability coefficient values may be the time constraint during testing. In the visual memory subscale, participants are required to memorize a target within 5 seconds and then identify the target within 20 seconds. People with schizophrenia show deficits in visual perception and require more time for visual processing [24,25]. Further studies are warranted to evaluate whether not timing or giving more time for the visual memory subscale could enhance the test-retest reliability and internal consistency.

In this study, the MDC values at the 95%, 90%, and 80% confidence levels in the overall scale and subscales of the TVPS-4 were provided. These values can assist clinicians and researchers when interpreting score changes (improvement or deterioration) in visual perception more reasonably. For example, a score change for an individual with schizophrenia exceeding the MDC_95_ (16.3) of the overall scale can be explained as a real change with 95% certainty. Substantial random measurement errors in two subscales (i.e., visual discrimination and form constancy, MDC% >  30%) were observed and the other subscales had MDC% of 26.1%–29.7%, which was close to 30%. The reason behind the substantial random measurement errors may be due to the long administration time which could result in fatigue and lead to a tendency of guessing answers. In this study, participants took 30–40 minutes to complete the seven subscales of the TVPS-4. The MDC% value was estimated at the 95% confidence level, which is more robust. People with schizophrenia may not achieve a real change with 95% certainty in the subscales. The MDC values at the other confidence levels (i.e., 90% and 80%) were estimated in this study. Future users could consider which confidence level they would like to assert to explain the score change for an individual with schizophrenia over two assessments.

Small effect sizes were observed, and the paired *t*-tests revealed significant differences between the two assessments in three subscales: visual discrimination, visual memory, and visual figure-ground. These findings suggest the presence of small practice effects in these three subscales. The results indicate that participants in this study may have easily memorized the answers or developed strategies to identify them. Two possible methods to reduce the practice effects are as follows: (1) repeating similar items more times to reach a plateau phase in the practice effect [26]; and (2) developing a computerized adaptive test, which can decrease the need to administer the same item in repeated assessments. Moreover, a computerized adaptive test can shorten the administration time which may improve the test-retest reliability of the TVPS-4. Future studies could consider developing a computerized adaptive test of visual perception to assess people with schizophrenia in a more precise manner.

This study had two limitations. First, we used a convenience sample recruited from a psychiatric center in northern Taiwan, which may restrict the generalizability of our results. Second, we did not examine the validity (e.g., construct validity with larger sample size), limiting the explanation of the visual perceptual constructs of the TVPS-4. Further validation is needed to confirm the construct validity of the TVPS-4 in people with schizophrenia.

## Conclusions

The TVPS-4 demonstrates acceptable test-retest reliability, satisfactory internal consistency, and trivial to small practice effect in people with schizophrenia. The overall scale is useful for assessing general visual perception and tracking progress over the course of intervention. However, two subscales revealed substantial random measurement error and three subscales demonstrated small practice effects. These subscales should be used cautiously when explaining specific visual perception abilities in clinical and research settings.

## Supporting information

S1 File Score of subtests. (XLSX)
